# Different pathogenesis of SARS-CoV-2 Omicron variant in wild-type laboratory mice and hamsters

**DOI:** 10.1038/s41392-022-00930-2

**Published:** 2022-02-25

**Authors:** Ya-Nan Zhang, Zhe-Rui Zhang, Hong-Qing Zhang, Na Li, Qiu-Yan Zhang, Xiao-Dan Li, Cheng-Lin Deng, Fei Deng, Shuo Shen, Bing Zhu, Bo Zhang

**Affiliations:** 1 Virus Laboratory, Institute of Pediatrics, Guangzhou Women and Children’s Medical Center, Guangzhou Medical University, Guangzhou, 510623 China; 2 Key Laboratory of Special Pathogens and Biosafety, Wuhan Institute of Virology, Center for Biosafety Mega-Science, Chinese Academy of Sciences, Wuhan, 430071 China; 3 University of Chinese Academy of Sciences, Beijing, 100049 China; 4 Hunan Normal University, School of Medicine, Changsha, 410081 China; 5 Wuhan Institute of Biological Products Co. Ltd, Wuhan, 420115 China

**Keywords:** Infection, Disease model

**Dear Editor**,

The COVID-19 pandemic caused by severe acute respiratory syndrome coronavirus 2 (SARS-CoV-2) continues to disrupt global public health since its first report in December 2019, resulting in more than 380 billion confirmed cases including nearly 5.7 billion deaths worldwide (https://covid19.who.int). Like other RNA viruses, SARS-CoV-2 evolves rapidly with accumulation of mutations in viral genome, giving rise to multiple variants and variants of concern (VOC), including Alpha (B.1.1.7 by Pango nomenclature), Beta (B.1.351), Gamma (P.1), Delta (B.1.617.2) as well as the newly emerged variant Omicron (B.1.1.529)^1,2^. The Omicron variant was first detected in November 2021 with substantial mutations relative to the original wild type (WT) SARS-CoV-2 strain (WIV04), including more than 30 mutations within the viral spike (S) protein that interacts with human angiotensin-converting enzyme II (hACE2) receptor for viral entry and infection. Due to its extremely high transmission rate, this variant rapidly outcompetes all the previous strains and becomes globally dominant responsible for the recent soaring COVID-19 cases. Numerous studies have indicated that fifteen mutations in the receptor binding domain (RBD) of the Omicron S protein, a region involved in direct interaction with the host receptor ACE2, confer resistance to current therapeutic mAbs or neutralizing antibodies elicited by previous infections or vaccines^3^. Besides, the binding of Omicron RBD to mouse ACE2 (mACE2) has also been reported recently^4^, indicating possible cross-species infection of Omicron for mice. Such new features of this variant thus raise some significant challenges, such as development of Omicron-specific therapeutic monoclonal antibodies (mAbs)/vaccines and establishment of reliable animal models for Omicron variant infection and pathology. Here, we investigated the pathogenicity of SARS-CoV-2 Omicron variant in normal wild type (WT) laboratory mice and golden (Syrian) hamsters, two key rodent models for SARS-CoV-2 infection and pathogenesis studies. We found Omicron variant could replicate efficiently in both young and old mice but without causing apparent disease symptoms. In contrast, the hamster is more sensitive to Omicron infection with apparent pathogenic damage in lungs lobes.

First, we assessed whether SARS-CoV-2 Omicron variant could use mACE2 as an entry receptor. To achieve this, BHK-21 cells that express mACE2 or hACE2 were used to evaluate viral infectivity. Briefly, BHK-21 cells were transfected with pCAGGS-mACE2 or pCAGGS-hACE2 expression plasmid 24 h prior to WT SARS-CoV-2 (WIV04) or Omicron variant infection at a multiplicity of infection (MOI) of 0.01. Different with WIV04 that was replicative only in hACE2-transfected BHK-21 cells, Omicron variant infection produced SARS-CoV-2 nucleocapsid protein (NP)-positive cells in both hACE2 and mACE2 transfected cells through indirect immunofluorescence assay (IFA) with SARS-CoV-2 NP specific antibody (Fig. 1a). The mACE2 or hACE2 expression in transfected BHK-21 cells was confirmed by IFA with S-tag antibody. As a negative control, there were no viral antigen-positive cells observed in naïve BHK-21 cells infected with WIV04 or Omicron variant. Our results demonstrated that SARS-CoV-2 Omicron could also use mACE2 as the entry receptor for viral infection besides the common hACE2 receptor used by other variants.

**Fig. 1:**
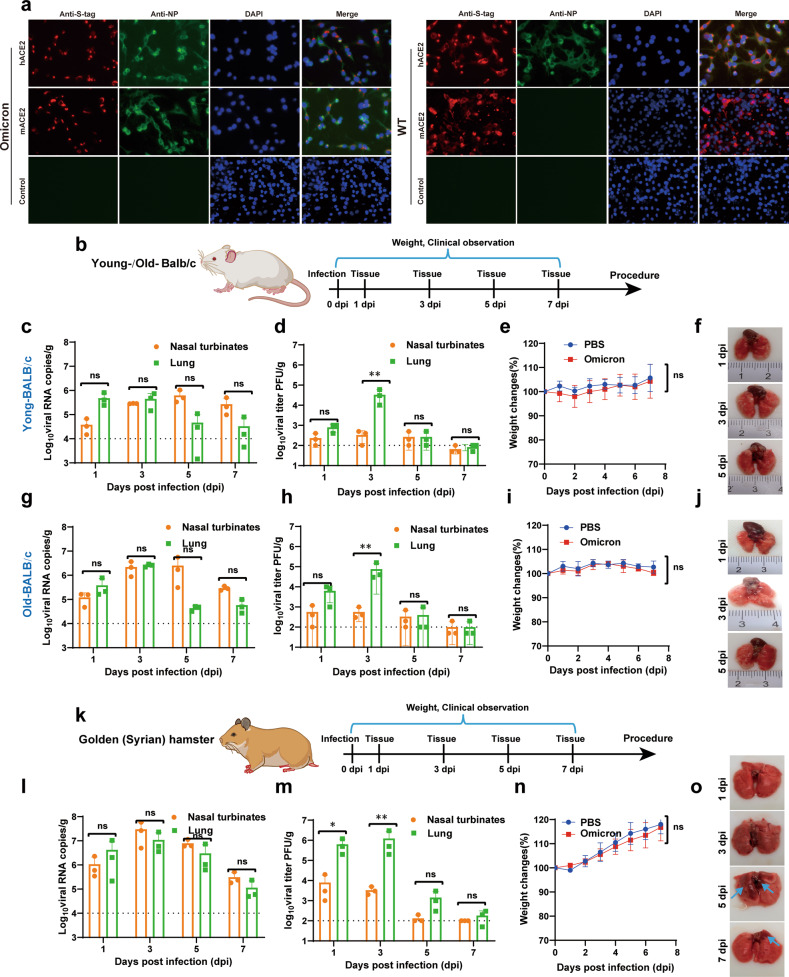
The pathogenesis of SARS-CoV-2 Omicron variant in WT laboratory mice and golden (Syrian) hamsters. **a** Immunofluorescence assay in BHK-21 cells expressing mACE2 or hACE2 infected with SARS-CoV-2 Omicron variant. BHK-21 cells were transfected with mACE2 or hACE2 expression plasmid. After 24 h post-transfection, the cells were infected with SARS-CoV-2 WT or Omicron variant at an MOI of 0.01. Cells were fixed at 24 hpi and stained for IFA assay to detect the expression of S-tag and viral NP proteins. **b** Flow chart of both young (8-week-old) and old (9-month-old) BALB/c mice (*n* = 3) infected with SARS-CoV-2 Omicron variant. All the BALB/c mice were intranasally infected with 2 × 10^4^ PFU Omicron variant in a total volume of 50 μL. **c**–**f** Characterization of SARS-CoV-2 Omicron variant infection in 8-week-old BALB/c mice. **c** Viral RNA loads and (d) virus titers of lungs and nasal turbinates were determined by qRT-PCR and plaque assays, respectively. **e** Body weight was monitored daily after the infection. **f** Pathological changes in the lungs of infected young BALB/c mice. **g**–**j** Characterization of SARS-CoV-2 Omicron variant infection in 9-month-old BALB/c mice. **g** Viral RNA loads and **h** viral titers were determined as above described. **i** Body weight was monitored daily after the infection. **j** Pathological changes in the lungs of infected old BALB/c mice. **k** Flow chart of SARS-CoV-2 Omicron variant infection in golden (Syrian) hamsters. The hamsters (*n* = 3) were intranasally infected with 2 × 10^4^ PFU Omicron variant in a total volume of 100 μL. **l**–**o** Characterization of SARS-CoV-2 Omicron variant infection in golden (Syrian) hamsters. **l** Viral RNA loads and **m** viral titers of lungs and nasal turbinates were determined by qRT-PCR and plaque assays, respectively. **n** Body weight was monitored daily after the infection. **o** Pathological changes in the lungs of infected hamsters. Obvious gross pathology changes in the lungs were depicted by blue arrows. Two-way ANOVA was used to determine statistical differences between groups. *p < 0.05, **p < 0.01, ns represents not significant

We then tested whether SARS-CoV-2 Omicron variant could infect laboratory wild-type mouse. Both old (9-month-old) and young (8-week-old) BALB/c mice were infected intranasally with 2 × 10^4^ plaque forming unit (PFU) Omicron variant in a volume of 50 μL DMEM. Mice from each group were monitored and weighted daily for 7 days (Fig. 1b). No obvious clinical symptoms and body weight loss were observed in infected mice from both groups (Fig. 1e, i). The viral replication dynamics in the lungs and nasal turbinates of mice were determined through quantification of viral genomic RNAs and infectious virus particles using real-time RT-PCR and plaque assays, respectively. The viral RNAs in the lungs of both old mice (10^6^–10^7^ copies/g) and young mice (10–10^6^ copies/g) increased from 1 day to 3 days post infection (dpi), and began to decrease from 5 to 7 dpi (Fig. 1c, g). Similar viral RNAs kinetic changes in the nasal turbinates were also observed in both groups of mice (Fig. 1c, g). Additionally, infectious viruses recovered from the lungs and nasal turbinates at 1, 3, 5, 7 dpi were confirmed by plaque assay, and the highest viral titers were observed in the lungs at 3 dpi (Fig. 1d, h). Although the overall virus yields in old mice, quantified by either real-time RT-PCR or plaque assay, were higher than those of young mice, there were no apparent gross pathological changes in all the lungs of Omicron variant infected mice from each group (Fig. 1f, j) at each time point.

Despite capable of replicating efficiently within lungs and nasal turbinates of infected mice, Omicron variant was not pathogenic for both old and young BALB/c mice. We further examined the pathological effect of Omicron in golden (Syrian) hamster. 4-to 5-week-old hamsters were infected with 2 × 10^4^ PFU Omicron variant in a total volume of 100 μL DMEM through intranasal route and were monitored and weighted daily for 7 days (Fig. 1k). All infected hamsters survived infection without apparent loss of body weights compared with mock infected group (Fig. 1n). The viral loads in lungs and nasal turbinates were quantified through plaque and quantitative real-time RT-PCR (qRT-PCR) assays at 1, 3, 5 and 7 dpi. SARS-CoV-2 Omicron variant could propagate efficiently in hamster as increased viral copy numbers were observed from 1 to 5 dpi (Fig. 1l). At the same time, infectious viral particles were also observed in lungs and nasal turbinates from 1 to 5 dpi and peaked at 3 dpi in the lungs (Fig. 1m). Importantly, the amount of infectious virus recovered from the lungs of hamsters (10^6^ PFU/g) was much higher than that of mice (10^3^ PFU/g versus 10^5^ PFU/g) at 1 and 3 dpi, which indicated that Omicron variant replicated more efficiently in hamsters than in mice. Additionally, there were obvious gross pathological changes in the lungs of infected hamsters at 5 dpi characterized by apparent focal or multifocal dark red discoloration in the lung lobes in contrast to homogeneously pink lung lobes observed at other time points (Fig. 1o). Overall, our results demonstrated that SARS-CoV-2 Omicron variant is more pathogenic to hamsters than mice. In the two very recent studies on SARS-CoV-2 Omicron variant in hamsters, there is some inconsistency regarding the replication capacity and pathogenicity in hamsters despite both demonstrated less pathogenic in Omicron-infected hamsters in comparison with other variant/strain. One showed that the omicron variant was not able to replicate efficiently in the lower respiratory tract of hamsters^5^. The other got the same conclusion as we have, that the omicron variant was replicative in both lungs and nasal turbinates, leading to lung damage that could be recovered later^6^.

Collectively, SARS-CoV-2 Omicron variant could utilize mACE2 as the receptor for viral entry and infection in common laboratory mice. Although SARS-CoV-2 Omicron is not pathogenic in mice, the common laboratory mouse model still could be used to evaluate efficacy of vaccine and antiviral therapy in some cases as this variant could replicate efficiently in the lungs and nasal turbinates of infected mice. Since the hamster is more susceptible to Omicron variant infection compared with the mice, it provides an important model recapitulating human infection and disease that helps better understanding of the pathogenesis of Omicron variant and improves rapid development of Omicron or even pan-variant targeted therapies.

## Supplementary information

Different pathogenesis of SARS-CoV-2 Omicron variant in wild-type laboratory mice and hamsters

## Data Availability

All data supporting the findings of this study are available within the article or from the corresponding author upon reasonable request.
