# Supplementary material for: Different pathogenesis of SARS-CoV-2 Omicron variant in wild-type laboratory mice and hamsters
Source: Signal Transduct Target Ther. 2022 Feb 25;7:62. doi: 10.1038/s41392-022-00930-2 (PMC8873353; doi:10.1038/s41392-022-00930-2)
Supplement: Supplementary file 1 — Different pathogenesis of SARS-CoV-2 Omicron variant in wild-type laboratory mice and hamsters [file 41392_2022_930_MOESM1_ESM.docx]

Supplementary Materials for

Different pathogenesis of SARS-CoV-2 Omicron variant in wild-type laboratory mice and hamsters

Ya-Nan Zhang^1,2†^, Zhe-Rui Zhang^2,3†^, Hong-Qing Zhang^2,3†^, Na Li^2,3^, Qiu-Yan Zhang^2^, Xiao-Dan Li^4^, Cheng-Lin Deng^2^, Fei Deng^2,3^, Shuo Shen^5^, Bing Zhu^1 *^, Bo Zhang^2, 3*^

^1^ Guangzhou Institute of Pediatrics, Guangzhou Women and Children's Medical Center, Guangzhou Medical University, Guangzhou, 510623, China

^2^ Key Laboratory of Special Pathogens and Biosafety, Wuhan Institute of Virology, Center for Biosafety Mega-Science, Chinese Academy of Sciences, Wuhan, China;

^3^ University of Chinese Academy of Sciences, Beijing 100049, China;

^4^ Hunan Normal University, School of Medicine, Changsha, 410081, China

^5^ Wuhan Institute of Biological Products Co. Ltd., Wuhan, 420115, China

†These authors contributed equally to this work.

*Correspondence to: Bo Zhang ([zhangbo@wh.iov.cn](mailto:zhangbo@wh.iov.cn)) or Bing Zhu (zhubing@gzhmu.edu.cn)

**This PDF file includes:**

Materials and Methods

**Materials and methods**

**Cell lines, viruses and antibodies**

Vero-E6 cells (ATCC CRL-1686) and Baby hamster kidney (BHK-21) cells were maintained in Dulbecco’s modified Eagle’s medium (DMEM, Gibco) supplied with 10% (v/v) fetal bovine serum (FBS, Gibco), 100 U/mL penicillin and 100 μg/mL streptomycin (Beyotime). The Omicron variant (CCPM-B-V-049-2112-18) was isolated from pharyngeal swab of infected patients in Hong Kong by the Institute of Experimental Animals, Chinese Academy of Medical Sciences. SARS-CoV-2 strain (WIV04) was originally isolated from a COVID-19 patient in Wuhan. SARS-CoV-2 wild type strain and the Omicron variant were propagated and titrated by plaque assays using Vero-E6 cells. All obtained viruses were stored aliquots at −80℃ and sequenced for experiments. The rabbit polyclonal antibody against SARSr-CoV Rp3 N protein and the mouse monoclonal antibody against S-tag for hACE2/mACE2 detection were kindly provided by Prof. Bing Yan at Wuhan Institute of Virology. FITC-conjugated goat anti-rabbit IgG and Alexa Fluor 568-anti mouse IgG were purchased from Proteintech, China.

**Plaque assay.**

Virus titration of SARS-CoV-2 was performed by monolayer plaque assay as described previously. Briefly, 1×10^5^ Vero-E6 cells were seeded into 24-well plate and then incubated with serially 10-fold diluted SARS-CoV-2 supernatants for 1 h at 37℃. After inoculation, the virus supernatants were removed and the monolayers were overlaid with a DMEM mixture consisting of 1% methylcellulose, 2% FBS, 100 U/ml penicillin and 100 μg/ml streptomycin. The cells were fixed with 3.7% formaldehyde for 24 h after 4-5 days incubation and stained with 1% crystal violet in water. Plaques were counted after washing with running water.

**Transient transfection and indirect immunofluorescence (IFA) assay.**

BHK-21 cells were seeded on a Chamber Slide (Nalge Nunc) in 6-well plate. After growing for 1 day, the cells were transfected with 2 μg plasmid DNA expressing mACE2 or hACE2 as manufacturer's Instructions (Promega Corporation), followed by infection with SARS-CoV-2 WT or Omicron variant at an MOI of 0.01 at 24 hpt. At the indicated time points, the cells were fixed with cold (−20 °C) 5% acetone in methanol at room temperature for 10 min, washed three times with PBS and fixed with 3.7% formaldehyde for 24 h. For the detection of viral replication, the cells were incubated with the antibodies against SARSr-CoV Rp3 N protein and S-tag for 1 h. After washing with PBS three times, the cells were incubated with FITC-conjugated goat anti-rabbit IgG and Alexa Fluor 568-anti mouse IgG at room temperature for 1 h. The nuclei were stained with DAPI. Following PBS washing, the slides were mounted with 95% glycerol and analyzed under a Zeiss fluorescence microscope.

**RNA extraction and quantitative real-time PCR (qRT-PCR).**

The lungs and nasal turbinates of mice and hamsters were homogenized in DMEM media and centrifugated. Viral RNA was extracted following the manufacturer’s protocol provided by QIAamp viral RNA mini kit (52906, Qiagen). qRT-PCR were performed using Luna® Universal Probe One-Step RT-PCR Kit (E3006). The following primer pair and probe based on SARS-CoV-2 S gene were used for the assay:

RBD-qF1: 5’- CAATGGTTTAACAGGCACAGG-3’

RBD-qR1:5’-CTCAAGTGTCTGTGGATCACG-3’

Probe: ACAGCATCAGTAGTGTCAGCAATGTCTC

**Animal infection experiment**

Two kinds of animal models were used in this study: BALB/c mice and golden (Syrian) hamsters. Both old (9-month-old) and young (8-/10-week-old) female mice were infected intranasally with 2×10^4^ PFU SARS-CoV-2 Omicron variant in a total volume of 50 μL; 4-5-week-old female golden (Syrian) hamsters were infected intranasally with 2×10^4^ PFU Omicron variant in a total volume of 100 μL. Mice and hamsters from each group were monitored and weighted daily for 7 days. Meanwhile, animals were sacrificed at 1, 3, 5 and 7 days post infection, and the lungs and nasal turbinates were collected for viral titration and viral genomic RNA quantification.

**Statistical Analysis**

All data were analyzed using GraphPadPrism 8.0.2 software and expressed as mean ± standard deviation (SD). The statistical significance was assigned when P values were < 0.05. Student’s T-test was used to analyze the differences between two groups, and significant differences between groups were determined using a one-way or two-way analysis of variance (ANOVA).
